# A qualitative study exploring the perceived effects of veterinarians' mental health on provision of care

**DOI:** 10.3389/fvets.2023.1064932

**Published:** 2023-02-07

**Authors:** Megan Campbell, Briana N. M. Hagen, Basem Gohar, Jeffrey Wichtel, Andria Jones-Bitton

**Affiliations:** ^1^Department of Population Medicine, The University of Guelph, Guelph, ON, Canada; ^2^Centre for Research in Occupational Safety and Health, Sudbury, ON, Canada; ^3^Ontario Veterinary College, University of Guelph, Guelph, ON, Canada

**Keywords:** veterinary mental health, stress, mental health, provision of care, veterinarian

## Abstract

**Introduction:**

Veterinary medicine is a rewarding, yet demanding profession with a myriad of occupational stressors that can impact the mental health of veterinarians. Stress, mental health outcomes, and associated risk factors amongst veterinarians have been well-researched. Much less research has investigated how high stress and/or poor mental health can impact veterinarians' provision of care.

**Methods:**

One-on-one research interviews were conducted with 25 veterinarians at a Canadian veterinary conference in July 2016 and verbatim transcripts were produced from the audio recordings. The research team collaboratively analyzed the interviews using thematic analysis.

**Results:**

Five themes described the perceived impacts of high stress and/or poor mental health: perceived negative impacts on interactions with (1) co-workers and (2) clients; (3) reduced concentration; (4) difficulty in decision making; and (5) reduced quality of care.

**Discussion:**

These results highlight the perceived impacts of self-reported high stress and/or poor mental health on veterinary team dynamics, the potential to impact case outcomes, and possibly endanger patient safety. Interventions to help mitigate the impacts of high stress and poor mental health are discussed.

## Introduction

Veterinarians are reportedly at a higher risk of experiencing depression, anxiety, stress, and/or suicidal ideations compared to the general population in Canada (1), the United Kingdom (UK) (2), the United States (3), Australia (4), New Zealand (5), Finland (6), and Germany (7). Multiple studies have described risk factors for poor veterinarian mental health (3, 7–9) and/or proposed interventions to improve the veterinary community's mental wellbeing (10–14). Yet, few published studies explore the impacts of mental health on veterinarians' provision of care (15, 16).

Compared to veterinarians (15, 16), there is considerably more research evaluating the impact of physicians' mental health upon their provision of care (17–25). This disparity in research is surprising given the commonalities the two professions share, including: both being responsible for improving the health of their recipients of care (26), both needing to successfully communicate with clients and/or patients (27–29), both being exposed to occupational stressors like long working hours (3, 6, 30–36), and both experiencing stress from angry patients or clients (37, 38). Thus, an overview of the impacts of physician and medical residents' mental health could be reasonably considered when evaluating the under-researched impacts of veterinarians' mental health on provision of care.

Burnout is reportedly associated with lower physician and medical resident perceived patient safety, more frequent perceived medical mistakes, lower perceived quality of care, decreased patient satisfaction, and reduced caregiver empathy (18–23, 39, 40). Furthermore, both stress and depression have been associated with medical residents making perceived medical mistakes in cross-sectional (39, 41) and longitudinal studies (22–24, 42, 43). Stress and depression have also been reported to impact the physician-patient relationship. For example, in a sample of 225 doctors in the UK, some participants noted that their depression resulted in short-temperedness, decreased concentration, and increased irritability with patients (41). A study of medical residents reported that higher perceived stress was positively correlated with higher burnout and both stress and burnout were significantly associated with decreased empathy over the course of a work shift (25). In turn, lower empathy predicted decreased patient-centered care (25).

To our knowledge, only two published studies have evaluated the impact of veterinarians' mental health on the provision of veterinary care. The impacts of depression, anxiety, stress, and burnout on client satisfaction were evaluated in veterinarians practicing in Ontario, Canada (16). Veterinarians with the highest and lowest levels of depression, anxiety, stress, and emotional exhaustion and depersonalization (two components of burnout) had higher client satisfaction compared to veterinarians who scored in the middle for these mental health outcomes. Empathy may help explain the counter-intuitive results (16). In human medicine, empathy has been positively associated with patient satisfaction (44), but may also make caregivers more susceptible to burnout (45). In the second study, veterinarians in Australia reported the perceived impacts of stress to include pre-occupation and self-doubt that hindered veterinarians' ability to concentrate (15). This is concerning for obvious reasons, including that concentration is necessary to ensure effective interactions and collaborative decision-making between veterinarians and clients (46).

Given the adverse effects of poor physician mental health upon patient care (18–23, 39–43), the elevated risks of poor mental health in veterinarians (1–3), and the paucity of published research on the impact of stress and poor mental health on provision of veterinarians' care, the objective of this study was to explore veterinarians' perceptions of the impact of perceived high stress and/or poor mental health on their provision of care (i.e., the care expected to be provided by a competent veterinarian serving the community) (47).

## Materials and methods

This study was conducted with a phenomenological approach within a constructivist paradigm (48). A phenomenological approach investigates how a phenomenon is consciously experienced by an individual (49). The constructivist paradigm understands that numerous interpretations of the same phenomenon can exist and that meaning is constructed through interactions with our surroundings (48, 50). A qualitative methodology was chosen for this study because of the wealth of perspectives and experiences that can be garnered when exploring under-researched topics (51), including how veterinarians' mental health impacts their provision of care.

### Data collection

One-on-one research interviews were conducted in a private conference room at the Canadian Veterinary Medical Association Conference in Niagara Falls, Ontario, Canada, from July 7–10^th^, 2016. Newsletters and emails from provincial and national organizations, as well as flyers sent through Veterinary Purchasing Ltd. (a major animal health product distribution company) were used to recruit participants. The Ontario Veterinary College communications team and researchers also shared recruitment details *via* social media.

Eligible participants were Doctor of Veterinary Medicine (DVM) degree holders or equivalent and who were able to communicate in English. An *a priori* sample size of 25 veterinarians was chosen for logistical reasons and based on comparable sample sizes in other veterinary mental health studies (15, 52). Data collection ceased when the 25 interviews were completed, as the interviewers were satisfied that no new participant information was being collected (53, 54).

The interviews were conducted using a semi-structured interview guide and were 24–90 min in length (average: 57 min). The interviews were audio-recorded and transcribed verbatim and transcripts were checked for accuracy by the primary author (MC) (55). The interview guide included questions to gain insights into veterinarians' lived experiences of mental health and potential impacts of mental health on veterinarians' work; only the data pertaining to the latter are reported here. Participants also completed a short demographic questionnaire.

### Data analysis

Braun and Clarke's approach to thematic analysis was used to guide the data analyses (56). Here, the primary author (MC) familiarized herself with the transcripts to explore preliminary patterns across the data set (56). MC selected three transcripts and open-coded the data to construct a first draft codebook. The interviews were inductively coded, meaning the codes were developed from the raw data rather than having pre-established codes that have been devised from theoretical frameworks or the published literature (57). BNMH and AJB independently coded the same three transcripts using the codebook. Using the analysts' feedback, MC then modified the first draft codebook to produce the working codebook (58).

Throughout the analysis stage, the codebook was modified by adding, separating, or merging codes, until no new codes emerged in the data, producing the final codebook (58). At least two members of the analysis team (AJB, BNMH, MC) independently analyzed the remaining transcripts using the codebook. Once completed, the analysis team collaboratively sorted the codes into major themes (56). The researchers subsequently evaluated, refined, described, and named the themes (56). The connections between various codes and themes was ascertained using thematic mapping (56). Quirkos^®^ v.2 software (59) helped facilitate the qualitative data analysis, while descriptive statistics were calculated for the demographic data using STATA^®^ v.16 software (60).

Numerous techniques were used to help establish rigor (61). Member checking occurred at the end of each interview, when the interviewer provided a summary to each participant and sought clarification as needed (62, 63) to promote reliability (61). Credibility was established by having peer debriefings and regularly scheduled meetings throughout the analysis stage to discuss research members' interpretations of the data (61–63). Negative case findings/disconfirming evidence (i.e., data that does not fit the emerging themes) were incorporated into the results to help promote validity (57, 61). An audit trail enhanced dependability by recording why the researchers inserted, merged, and/or deleted codes or themes (64, 65). Finally, transparency was strengthened by the primary author using a reflexivity journal during the analysis and writing stages to reflect on how her own thoughts about the research evolved throughout the analysis (61). Verbatim quotes are provided for context and to support findings (66). The Consolidated Criteria for Reporting Qualitative Research (COREQ) checklist helped guide the reporting of this study (67).

### Ethical considerations

The study protocol was approved by the University of Guelph Ethics Board (REB 16MY029). Every participant gave written, informed consent prior to participation. At the conclusion of each interview, each participant received an $100 Canadian honorarium. Potentially identifying data, such as names, dates, and places were removed from the dataset after transcription to uphold participant confidentiality.

## Results

A summary of participants' demographic data is presented in Table 1. Most participants identified as women (20/25; 80%), were married (21/25; 84%), small animal veterinarians (19/25; 76%) and were associate veterinarians (13/25; 52%). The mean age of the participants was 49.7 years. Veterinarians interviewed were diverse and had a range of roles, ages, and experience levels. As noted in Table 1, a small number of participants were no longer in clinical practice; however, these participants often answered questions from their time in clinical practice.

**Table 1:** The demographic factors of veterinarians (*n* = 25) who participated in one-on-one interviews.

**Variable**	**Sub-categories**	**n**	**Percentage**	**Range**
**Gender**				
	Women	20	80.0%	-
	Men	5	20.0%	
**Age**				31–69 years
	30–40	7	28.0%	-
	41–50	4	16.0%	-
	51–60	9	36.0%	-
	61–70	5	20.0%	-
**Type of Medicine Practiced** ^*^				
	Small animals	19	76.0%	-
	Equine/Bovine	4	16.0%	-
	Avian/Exotic	6	24.0%	-
	Small ruminant	1	4.0%	-
	Other^a^	6	24.0%	-
Relationship status				
	Married	21	84.0%	-
	Divorced	1	4.0%	-
	Committed relationship	2	8.0%	
	Single	1	4.0%	
**Salary**				$45,000-$200,000
	$45,000–$95,000	16	64.0%	-
	$96,00–$145,000	6	24.0%	-
	$146,000–$200,000	3	12.0%	-
**Role at clinic**				
	Associate	13	52.0%	-
	Owner	4	16.0%	-
	Other^b^	5	20.0%	-
	Missing Values	3	12.0%	-

^*^Values added up to more than 100%, since categories were not mutually exclusive.

^a^Other includes locum veterinarians, educators at veterinary institutions, and specialty veterinarians.

^b^Other includes academia, locum veterinarians, specialists, an owner who was becoming an associate, and unspecified.

Participants described numerous ways in which they perceived their experiences of high stress and/or poor mental health impacting their work and provision of care. The impacts were described within five themes: perceived negative impacts on interactions with (1) co-workers and (2) clients; (3) reduced concentration; (4) difficulty in decision-making; and (5) reduced quality of care. Each theme is described below.

### Theme 1: Perceived negative impacts on interactions with co-workers

Participants perceived their high stress and/or poor mental health to negatively affect interpersonal relationships with co-workers. Participants readily described being “snappier,” having a “short fuse,” having their “tone change,” or being “on edge” when encountering occupational stressors or when, for example, “my anxiety is bad.” These interactions with colleagues were said to put a strain on co-workers' relationships as it ‘changes the relationship' by altering ‘co-workers' perspective' and ‘trust in me', while also “making [the staff] upset” or “bring[ing] people down.” Several participants recognized that other veterinary staff, such as veterinary technicians or animal care assistants, “bear the brunt” of this irritability.

“*Okay, yeah, so when you're anxious, or tired, or exhausted, you're probably not, you're probably snappier at people, you have less patience for people, and so as a result, yeah, you could probably not be the nicest to your co-worker, like every day is fine, but then—like when you're with people for this long, you sort of become family and you take people for granted.”* (DVM 8)

Although acting in a “less kind” manner toward co-workers was the most common change in co-worker dynamics described, other participants described internalizing their feelings or undergoing social withdrawal when experiencing poor mental health. For example, one participant said they got “annoyed by everything,” yet “I often don't even want to like, point out things that technicians are doing that I'm getting annoyed by because I feel like it's not worth the trouble.” Social withdrawal was described by one participant as being “in my office by myself”:

“*…I don't always participate in, say, lunchtime discussions, or anything like that, when I'm in my depressed mode, I don't visit with the [staff] as often.”* (DVM 14)

In several instances, participants readily described the negative impact of high stress and poor mental health on co-worker interactions to extend broadly to negatively impact “the energy of the clinic” at-large. For example, participants recalled being able to “feel right away” the “tension” when co-workers were having “off days.” The “atmosphere” was also said to shift the moods of other colleagues working in the space.

“*In terms of how I can see it, is that you know, the [staff members] are you know just dragging through their procedures as well, you know they're not bubbly and enthusiastic and talking about other uh, things, you know normally, the chatter…So when that's not happening, you know when it's relatively silent, everybody is feeling the weight of this, uh, burden of emotion.”* (DVM 2)

“*But you can definitely tell when um, like, when somebody is having an off day, or they're not engaged, you know they're not working toward the same goal as you are, I guess. Then it's frustrating, you feel like you're lagging, and you should be doing 14 things, when you can only manage 10.”* (DVM 6)

Inversely, an atmosphere filled with “positivity” was described as a motivator and could help the staff to “handle things [at the practice].” A range of workplace energies and outcomes were described further:

“*…it's camaraderie in the clinic, or being able to bond with a team, and have the fun in the day without losing the efficiency at work…But it's at such a pace that you're productive, it's healthy, it's a positive environment, and I think that's when people do their best, is because they are open to ideas, they can think laterally, they have the energy to do the extra mile if you need.”* (DVM 15)

Indeed, feelings of wellbeing were said to “help me just interact with the staff better and foster a “supportive” workplace atmosphere.” One participant described their wellbeing as beneficial to colleagues who were trying to cope with occupational stressors endemic to the veterinary profession. The veterinarian attested:

“*I think that my wellness has been, I hope anyway, a real positive influence on all my employees and many of my clients. I think my overall attitude toward life and so forth is one that has helped—well I know it's helped other vets because they've told me so, in dealing with some of the stresses and challenges of work.”* (DVM 11)

### Theme 2: Perceived negative impacts on interactions with clients

Participants readily described their high stress and/or poor mental health impacting “how I talk with clients” and as contributors to a reduction in effort invested in client interactions. For example, one participant recalled: “I like die a little inside, and I don't want to answer [clients] questions” and another reported “[not] feel[ing] like necessarily giving all the advice.” Others “don't try as hard to try and convince the [client],” as they 'just don't feel like dealing with the bite back of the owner disagreeing.” Being “short” or “less patient” when communicating with clients were other impacts of veterinarians experiencing poor mental health. Participants noted:

“*Whereas, by the end of the day, I have the mentality of ‘do whatever you want it's not my problem', and like, I just need to get through the rest of the day, I don't want to have this discussion, and I feel like … you know whether or not that conversation is going to make a difference anyways, I'm not necessarily giving the best advice I should be, because mentally I'm just done with dealing with that.”* (DVM 17)

“*There's nothing left…Oh, it was not good, I'm not good at [interacting with clients]. Because I didn't have the strength to have compassion, if compassion was needed.”* (DVM 15)

The implications of the ineffective communication with clients were described as altering “client's perspective and trust in me” and even loss of clients:

“*Yeah, so if you're obviously coming to work stressed you're probably not as interactive with your clients, so you may leave, you may not explain fully to a client what is going on with their animal, and then they leave confused and they may never come back to see you again because they've just wasted money and they still don't know what is wrong with their animal.”* (DVM 8)

A few participants described feeling like their clients were cognizant of their veterinarian's poor mental health through non-verbal communication, like “body language.” One veterinarian shared:

“*Well yes, because if you know you're talking to someone that's depressed, they're not as lively in conversation as they might be otherwise. I mean you can…body language, by facial expression, by you know tiredness, by enthusiasm for world events, and uh, even town events come more into play, but if you're depressed and burdened with emotion, then you know you can see that in people.”* (DVM 2)

Other participants “didn't think” that their poor mental health had any effect on their client interactions. They described forcing “a cheery atmosphere with [clients]” when experiencing high stress and/or poor mental health, such as “turn[ing] [my emotions] off long enough to be able to do my job effectively” or making a conscious effort to “try not to let it” affect their client interactions. In the case of the latter, a veterinarian remarked:

“*But when I go in to see the client, I kind of like, ‘Okay, you know, you're going to go in, you're not going to let this affect your interaction with the client.' So, the last reserves go to that.”* (DVM 16)

Conversely, when participants described feeling well, they said that were more effective communicators, as they could “explain things better” or perceived that the clients “were understanding what I'm saying.” In addition, veterinarians said that they were more “engaged with the client,” thus prompting participants to “pick up on body language” or other “nonverbal cues.” Two veterinarians articulated:

“*When I'm not [feeling] burned out and I'm not angry, I am more patient with clients, and I will talk, um, I'll talk to them for a lot longer. I'm an introvert, so talking to people takes effort, but if I'm, if I'm in a better mood, it comes more naturally rather than if you have to drag it out of me, right? And I also tend to explain things better.”* (DVM 5)

“*[Well-being] impacts my work not in a great way anymore, but it definitely impacts the subtlety of my work, so how much I put into it and how much the client gets; not so much how much the animal gets, it's more the client interactions that I have. So if I'm feeling good, I will explain things better, I get handouts for clients, I do that extra stuff to make sure that I think they understand fully what's going on.”* (DVM 13)

### Theme 3: Reduced concentration

Veterinarians reported that their high stress and/or poor mental health negatively impacted their ability to concentrate. Attempting to focus when feeling anxious was depicted as difficult and thinking was “fuzzy.” Another veterinarian stated that her anxiety-induced inattention elevated her stress levels to the point where it made it “hard to function.” One participant articulated the effects of her poor mental health, stating,

“*Yeah, I was just, it was just really hard to concentrate on anything, hard to focus, I just constantly felt like I was going to throw up, and I was shaky, and I'm never, I'm not [normally] an anxious person, like I've never had that before, so. It was really hard for me to deal with that.”* (DVM 9)

The time and place of veterinarians' inattention differed amongst participants, with a few sharing that they could not focus “on anything” at work. Another acknowledged that her concentration only waned during her “down times” when her personal stress was not “checked at the door” before coming into work. She explained:

“*And on the days that yeah I don't check [the stress] as well, it's hard for me to, in those downtimes when I'm doing my own thing and not seeing clients, you know in the in-between time, I have trouble focusing and getting things done in a timely manner.”* (DVM 21)

In several instances, participants shared the implications of their lack of focus, including impeding patient care as “it takes more time” and more drastically, “mak[ing] mistakes [as] I probably was distracted by either what was going on in my head or what was going on around me.” Being “distracted” was also said to be extremely detrimental during time-sensitive veterinary surgeries and was said by one participant to result in fear of litigation. For example:

“*And when you're anxious, for example in surgery, if you're anxious and you always second guess yourself, you're always checking or you're not sure, and you're sort of hesitant, so then that takes more time. And so, as a result when you take more time in surgery and stuff like that, it's not necessarily better for the patient. I mean it's good to double check things and stuff like that, but when you're hesitant and you have that much self-doubt, it doesn't really translate to a good thing for your patients.”* (DVM 8)

“*But then if you're not mentally well, you're not focused, you're not concentrating on what you're supposed to do. If you're in a clinic setting it could be malpractice because if you're not, you know, if you're sitting in a surgery doing surgery on an animal and your mind is somewhere else and you're not even looking at what you're tying off, cutting, you know, you're in trouble.”* (DVM 10)

### Theme 4: Difficulty in decision-making

Participants readily described difficulty in making decisions when experiencing poor mental health and/or high stress. Many participants commented that they “second-guess” themselves or “perhaps you don't make decisions that are as good” because they felt “stressed” or were “not feeling well and at 100%.” The largest consequence of “double-think[ing] everything” was said to be “you don't feel as confident in your decisions.” One participant illuminated:

“…*when I'm feeling stressed, I think I feel, my decision-making just feels disjointed, I guess. I think that's the best word I can think of. So that I think I double-think everything, like I don't trust my judgment, and so then I revisit it, I guess, I think I spend more time spinning my wheels.”* (DVM 23)

Poor mental health leading to second-guessing was described as a “vicious cycle” that fed itself. Unless someone intervened “to help you break the cycle” or “for you to realize there's an issue and seek help” then the cycle was described to persist, causing “[veterinarians to] continue to go further and further and further, deeper into depression, or deeper into self-doubt.” One veterinarian described “break[ing] this cycle” when he sought help for his poor mental health. He stated:

“*It allowed me the ability to make decisions again, because during that time before medication I wasn't really able to make effective decisions, but when I was on the meds I could, and that allowed the business to be saved, although we did have to let people go, and I still felt bad about that … It didn't depress me as much.”* (DVM 13)

### Theme 5: Reduced quality of care

Poor mental health outcomes, such as “stress” and being “depressed” were described to impact the quality of care that veterinarians provided. Some participants expressed an inability to “really fully assess everything about the [patient],” “doing a quick, short exam, just to get through it,” or not giving the patient “extra attention” as needed when the veterinarians were in a “bad frame of mind” or “absorbed in [their] own family trauma.” Other veterinarians felt that their poor mental health impacted their ability to think through cases, which in turn led to less attention directed toward the patient. One veterinarian elaborated:

“*Because if you're… not, uh, cognizant of everything you're doing, and thinking beyond, that is to say if you're trying to make a diagnosis for example, you've got to think outside of the box. You can't just look at the numbers on the page, you've got to take the patient and family situation, and where they've been in the last week or two. You've got to think of all of those things as a process. And if you're depressed and don't care, then you're going to miss stuff.”* (DVM 2)

Other participants described indirectly witnessing their quality-of-care fluctuate with the “ups and downs” of their colleagues' mental health. As one participant spoke about their colleague who was experiencing a mental health challenge:

“*Things would get dropped. And I'd look at cases and she just did the bare minimum. And then she'd have days where she was fantastic again. And we did bring it up, and that's when [my colleague] said, you know, “I'm struggling with this stress in my life…” Like we had a patient in hospital over the weekend, and was on three times a day antibiotics, and she decided it was okay just to do it twice, because that was when someone was coming into the building.”* (DVM 17)

## Discussion

This study used an exploratory qualitative approach to help address the gap in knowledge around the impacts of poor mental health on veterinarians' provision of care. Participants' perceptions of the impacts of high stress and/or poor mental health were evident in five themes: perceived negative impacts on interactions with (1) co-workers and (2) clients; (3) reduced concentration; (4) difficulty in decision-making; and (5) reduced quality of care.

Veterinarians in our study described being short tempered, irritable, or withdrawing from their co-workers when experiencing high stress and/or poor mental health. These findings mirror those reported among human healthcare providers (68) and participants in non-healthcare settings (69) experiencing poor mental health. Veterinary staff may be engaging in a cycle where unhealthy veterinary relationships contribute to poor veterinarian mental health (70) and our findings suggest that veterinarians' poor mental health further strains veterinary relationships.

Similar to previous physician research (41, 71) many participants in our study said that their high stress and/or poor mental health decreased the quality of their communication with clients, particularly the effort and patience invested in client interactions. While this was a commonly reported effect, other participants noted that their poor mental health had no effect on client interactions. This concurs with a human healthcare study where high physician burnout was not significantly correlated with patient-centeredness, patient satisfaction, or patient trust (72). Unexpected results were also observed with clinical veterinarians in Ontario, as respondents with poorer mental health scores (representing elevated stress, burnout, anxiety, depression, and secondary traumatic stress) received higher client satisfaction scores than veterinarians scoring in the mid-range for those measures (16). The latter study, as well as the present study findings, suggest that the relationship between veterinarian mental health and client interactions is unlikely to be straightforward. Interpretation of our study may also be hindered by participants' subjective reporting of how mental health impacts their care. In human medicine there are mixed results as to whether physicians' *perception* of giving substandard care equates to actually delivering substandard care to patients (21, 24, 40, 73). An incongruence between objective findings and physicians' subjective perceptions may be because physicians with poor mental health evaluate their performance more harshly than their peers or their patients (24, 74). Future studies could evaluate if veterinarians' mental health is associated with more critical self-evaluation of their provision of care.

Participants reported that high stress and/or poor mental health reduced concentration, negatively impacted decision-making, and increased the risk of making medical errors. These findings align with reports of high stress resulting in preoccupation amongst veterinarians in Australia (15) and the association between reduced veterinary concentration and medical mistakes in the United Kingdom (75). Indeed, impaired concentration is one of the most common cognitive implications of mood and anxiety-related disorders (76). Further concerning is that veterinarians may have a prolonged period of self-doubt, where they question their career choice, or even search for non-clinical veterinary jobs after making surgical mistakes (77).

Participants reported that their quality of care was also impacted by their poor mental health and/or high stress. The World Health Organization (WHO) has identified six quality of care domains (78); here, we emphasize the safety domain. Safety, in the context of healthcare providers, is defined as minimizing the risk and/or injury to patients (78). Our participants discussed overlooking potentially pertinent information, which could have detrimental impacts on the outcomes of cases and jeopardize patient safety.

### Recommendations

Our study has highlighted that veterinarians' poor mental health can adversely impact their relationships with co-workers, thus one way to foster positive relationships with colleagues may be through emotional intelligence (EI) training. EI is the ability to perceive, understand, and manage emotions in oneself and others (79). Understanding one's emotions in themselves and others can have positive life outcomes, such as helping individuals select a meaningful career and thrive in their job, maintaining physical health, and fostering good interpersonal relationships (80). In a study of pediatric staff in Israel, EI interventions were reported to improve interpersonal, intrapersonal, stress management, and adaptability scores compared to control populations without such training (81). Hence, the development of EI training programs geared toward all members of the veterinary team in a clinical setting may be advantageous for the relationships and mental wellbeing of veterinary employees. Given that provision of veterinary care is a team endeavor that requires good communication and trust between clients and amongst all veterinary staff (82, 83), the implementation of EI training may also have beneficial impacts on the quality of veterinary care provided. While some occupational stressors do not lend themselves easily to modifications, EI can help employees better recognize, understand, express, and regulate their emotions, which can help reduce personal and interpersonal tensions and better position team members to respond to those stressors.

Findings from our research indicate that poor mental health can reduce veterinarians' ability to concentrate. Veterinarians regular use of meditative practices could be helpful. One qualitative study reported that nurses who attended meditation style classes (i.e., yoga, Tai Chi, or Reiki sessions) self-described increased focus and attention and improved problem-solving abilities while at work (84). Veterinarians' inattention was also self-described to increase their susceptibility to making medical mistakes. Self-compassion training (85), cognitive reframing (86), and candid discussions with colleagues who understand and experience similar feelings of self-doubt or self-criticism (13) may all be useful responses to feelings of self-doubt and in response to making medical errors.

The potential for veterinarians' mental health to impact quality of care further highlights the need to implement organizational-level wellbeing interventions, as well as robust and systemic quality improvement measures in veterinary medicine. Quality improvement aims to refine the quality of veterinary medicine at a systematic level by identifying problems and implementing and measuring the effectiveness of interventions to address those problems (87). Empirical evidence suggests that specific controls, such as clinical audits and checklists can increase the quality of patient care (88) and significantly decrease errors (89) in veterinary settings, respectively. Yet, time constraints and colleagues' unsupportive attitude toward quality improvement measures may be deterrents for further widespread implementation (87). We propose further research into this topic to inform veterinary employers and employees on best practices for maintaining or improving veterinarians' quality of care and the potential protective effect on mental health. Furthermore, employers that are supportive of quality improvement measures could update their policies to make some of these tools (i.e., checklists for surgeries) mandatory within the veterinary clinic.

This study contributes to the understanding of the impacts of high stress and/or poor mental health on veterinarians' provision of care. A strength of this study is that there was a diversity of participants, including veterinarians with differing roles, ages, and experience levels. One limitation is that participants subjectively described the implications of their poor mental health upon their provision of care and these subjective experiences may not align with objective findings (24). The research interviews were also conducted prior to the beginning of the COVID-19 pandemic. Veterinarians' mental health and practices of veterinary medicine have likely changed due to the pandemic and these differences were not captured in our data.

## Conclusion

The results from this study suggest that high stress and/or poor mental health may have widespread and far-reaching impacts on some veterinarians' provision of care, including perceived negative impacts on interactions with (1) co-workers; (2) clients; (3) reduced concentration; (4) difficulty in decision making; and (5) reduced quality of care. This study also emphasizes that mental health may not be just an individual level (i.e., veterinarian) problem, but rather, may have clinic-wide impacts, including potentially ineffective client communication, strained colleague relationships, and jeopardizing patient safety. The results of this study call for additional wellbeing interventions and strategies to be implemented at the clinic/organizational levels to safeguard the mental health of veterinary staff and ensure that veterinarians' provision of care is not compromised due to high stress and/or poor mental health.

## Data availability statement

The original contributions presented in the study are included in the article/supplementary material, further inquiries can be directed to the corresponding author/s.

## Ethics statement

The studies involving human participants were reviewed and approved by University of Guelph Research Ethics Board. The patients/participants provided their written informed consent to participate in this study.

## Author contributions

AJ-B and CB recruited participants, constructed the interview guide, performed data collection, and developed and proposed the project. The qualitative interviews were checked for accuracy against the audio-recordings by MC. The qualitative interviews were jointly analyzed by MC, AJ-B, and BH. This paper was written by MC under direct supervision by AJ-B, in consultation with BH. BG and JW provided further feedback, editing, and interpretation for the manuscript. All authors contributed to the article and approved the submitted version.
